# Gender differences in the effect of medical humanities program on medical students’ empathy: a prospective longitudinal study

**DOI:** 10.1186/s12909-020-02333-9

**Published:** 2020-11-10

**Authors:** Michal Lwow, Laura Canetti, Mordechai Muszkat

**Affiliations:** 1 Department of Medicine, Hadassah Medical Center, Mount Scopus, POB 24035, 91240 Jerusalem, Israel; 2 Department of Psychology, Hebrew University of Jerusalem, Mount Scopus, 91905 Jerusalem, Israel; 3 Department of Psychiatry, Hadassah Medical Center, POB 24035, 91240 Jerusalem, Israel

**Keywords:** Empathy, Medical humanities, Admission system, Gender

## Abstract

**Background:**

Previous studies have suggested that Medical students’ empathy declines during medical school, especially during the clinical studies. The aim of this study was to examine. Changes in medical students’ empathy during their first clinical experience, and to determine the impact of gender and humanities curriculum on empathy changes.

**Methods:**

In this prospective longitudinal study, 262 4th year students from three consecutive classes were assessed. Empathy was assessed before and at 4th-year-end, using the Jefferson Scale of Physician Empathy-Student Version (JSPE-S). The three classes differed in humanities curriculum [limited Medical Humanities (MH_(lim)_) vs. extended Medical Humanities (MH_(ext)_)], and in admission system [Personal Interview (PI) vs. multiple mini interviews (MMI)].

**Results:**

Overall, there was a small but significant decrease in JSPE-S during the fourth year (114.40 ± 11.32 vs. 112.75 ± 14.19, *p* = 0.034). Among men there was a statistically significant decline in JSPE-S during the fourth year, and the MH_(ext)_ (but not the MH_(lim)_) was associated with the decline (*t*_(35)_ = 2.38, *p* = 0.023). Women students showed no decline in empathy during the fourth-year of studies, regardless of type of humanities program. In addition, women who participated in MH_(ext)_ had a higher JSPE-S scores during the 4th -year as compared to women who participated in MH_(lim)_.

**Conclusion:**

Pre-clinical humanities program was associated with a decline in empathy among men medical students during the fourth-year of medical studies. Gender differences in response to medical humanities programs require further study.

## Background

Enhancing physician’s empathy towards patients is recognized as an important aim of medical education [1–3]. Empathy is defined [3] as involving cognitive and emotional domains [4]. ‘The cognitive domain of empathy involves the ability to understand another person’s inner experiences and feelings and a capability to view the outside world from the other person’s perspective. The affective domain involves the capacity to enter into or join the experiences and feelings of another person’ [5].

Empathic patient-doctor communication can increase patients’ trust and satisfaction [6, 7], increase adherence to treatment [7, 8], and also reduce the number of legal claims against primary care physicians [9]. However, most of the studies on empathy changes during medical studies have suggested that empathy declines, rather than increases during studies [1, 10–12]. In a cross-sectional study of empathy among medical students, Chen et al. showed that first-year students had the highest empathy scores whereas the fourth-year students had the lowest scores [10]. Two longitudinal studies showed a decline in empathy during medical studies [1, 11]. Interestingly, most of the studies showing a decline in empathy during medical school have suggested that the decline is largest following students’ exposure to clinical life during clerkships [1, 10–12]. Reviews of studies reporting on empathy at various stages of physician training suggested that empathy declines during medical school and residency [13], however the decline was suggested to be small [14].

Studies have reported on higher empathy scores among women medical students as compared to men [1, 11, 15–17]. However, reports on the effect of gender on empathy changes among medical students’ have yielded inconsistent findings. While similar patterns of empathy decline have been reported in men and women in some studies [1, 11, 15], one study found that empathy declined between the third and the fourth-year of medical studies only in men but not in women [16].

Educational interventions have been suggested to prevent empathy decline during medical studies [18–23]. Previous studies have reported on higher empathy in women as compared to men, following such educational interventions [22, 23].

The main aim of the present study was to examine changes in medical students’ empathy during the first clinical year in medical school, and to determine whether gender and humanities curriculum moderate potential findings. An additional aim was to contribute to the validity evidence of the Hebrew version of the JSPE-S by examining its relationship with an established measure of empathy.

We hypothesized that students’ empathy will decline during their first clinical year of medical studies, and that an extensive 3-year preclinical medical humanities curriculum would prevent this decline.

## Methods

The study was approved by the ethical committee of Hadassah Medical School. Informed consent was signed by all participants.

### Context- structure of medical studies

The Hadassah Hebrew University of Jerusalem Medical School offers a six-year program. The first 3 years includes basic sciences and preclinical studies. During the first 3 years of studies, exposure to patients and everyday hospital life is limited and occasional. During the following 3 years, students attend hospitals or outpatient clinics in small groups on a daily basis. Thus, the first students’ significant clinical experience occurs at the fourth-year of studies.

### Study cohorts

The study included three consecutive cohorts, differing in humanities curriculum and admission system. The participation in the humanities program in each year was mandatory, thus each class was obligated to participate in the program offered in that year (limited/extended).

The first cohort (PI/MH_(lim)_) went through an admission process that included a 45-min panel-style personal interview. The second and the third cohorts (MMI/MH_(lim)_ and MMI/MH_(ext)_) went through a multiple OSCE-style mini interviews (MMI)-based admission process [24].

**Fig. 1:**
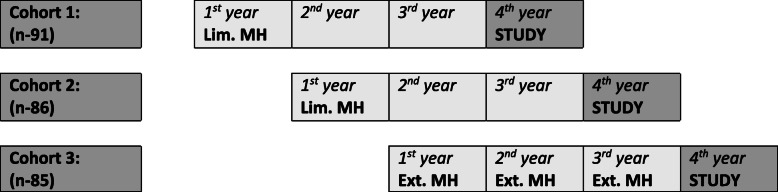
Study schedule and medical humanities program according to medical school year in the three study cohorts. Lim. MH: limited Medical Humanities program. Ext. MH: extended three-year Medical Humanities studies. 1^st^, 2^nd^ and 3^rd^ years of 6 year medical school program: limited clinical exposure. STUDY: During the 4^th^ year of studies, students filled the questionnaires twice: prior to clinical studies and 8 months later, at the end of the year

The three consecutive cohorts included (Fig. 1):

1. PI/MH_(lim)_ cohort: Personal Interview (PI), limited Medical Humanities program (MH_(lim)_) (*n* = 91).
2. MMI/MH_(lim)_ cohort: multiple mini interviews (MMI), (MH_(lim))_ (*n* = 86).
3. MMI/MH_(ext)_ cohort: MMI, extended Medical Humanities program (MH_ext_) (*n* = 85).

### Humanities program

The PI/MH_(lim)_ and the MMI/MH_(lim)_ cohorts included a pre-clinical humanities curriculum that was limited to the first year of medical school. The third cohort (MMI/MH_(ext)_) participated in an extensive and comprehensive three-year pre-clinical curriculum.

The extended pre-clinical humanities program was taught during the first 3 years of medical school, including the following courses:

During the first year of medical studies the program included the ‘Human and Medicine’ course on patient- doctor communication, cultural competency in medicine, basic principles of medical ethics, and physician professionalism. In addition, the first year course included early clinical exposure and community medicine. During the second year of medical studies the program included courses such as history of medicine, medicine and literature, physicians and holocaust, narrative medicine. During the third year of studies the program included the following courses: medicine and law, introduction to human sexuality and introduction to breaking bad news [25].

### Study population

Three hundred forty-two medical students consented to participate in the study. 264 (77%) of these provided full answered questionnaires on the beginning of the 4th year. Among these, two students had a repetitive filling pattern which wasn’t consistent with the content of the questionnaire and were excluded. Out of the remaining 262 medical students, 35 (13.4%) students did not fill the end of year questionnaires. Thus, 227 students were included in the longitudinal analyses.

### Instruments

#### Interpersonal reactivity index (IRI)

The IRI is a validated 28-item self-report measure consisting of four 7-items subscales, each tapping some aspect of the global concept of empathy. The Perspective-Taking scale assesses the tendency of spontaneously adopting the psychological point of view of others; the Fantasy scale taps respondents’ tendencies to identify with feelings and actions of fictitious characters in books, movies and plays. The Empathic Concern scale assesses “other oriented” feelings of sympathy and concern for unfortunate others, and the Personal Distress scale measures “self-oriented” feelings of personal anxiety and unease in tense interpersonal setting [4].

The Hebrew version of the IRI has been widely used in research in Israel [26–28]. In the present study the alpha Cronbach coefficient of internal reliability of the IRI was very good: α = 0.81.

#### Jefferson scale of physician empathy – student version (JSPE-S)

The JSPE-S was developed to measure empathy specifically within the context of the physician–patient relationship [5]. It includes 20 Likert scale items which are scored from 1 (strongly disagree) to 7 (strongly agree). The questionnaire was validated in numerous studies and is frequently used in medical education research and has been translated to more than 42 languages [1, 3, 29, 30]. The questionnaire encompasses 3 components of empathy; perspective taking (considered the core component of empathy), compassionate care and standing in the patient’s shoes [3, 5]. The English version of JSPE-S was translated to Hebrew by four physicians who speak English fluently, and was translated back to English by native English speakers who is also fluent in Hebrew (‘forward-backward’ procedure). Once the preliminary Hebrew version was obtained, the questionnaire was administered to 3 other physicians to achieve a consensus regarding its final version. We examined convergent validity with a similar instrument (the Interpersonal Reactivity Index (IRI).

#### Socio demographic questionnaire

Participants were asked to provide information regarding their gender, age, ethnicity, marital status, religiosity, and preferences regarding future residency.

### Procedure

The design of the study was longitudinal: investigators distributed questionnaires in two different time points to each cohort during the three study years. The JSPE-S, the IRI and the socio-demographic questionnaire were administered at the beginning of the fourth-year, before attending clerkships. The JSPE-S was distributed to students again at the end of the fourth-year. For all three cohorts the interval between the first and the second questionnaires was 8 months. All questionnaires were filled anonymously. Each participant received a random number, which was written on his/her questionnaire and was used to identify the individual pre-post questionnaires. Students were allowed to return the questionnaires during the following 7 days*.*

### Data analyses

One way and two-way ANOVAs and t-tests were used for continuous variables, and *χ*^*2*^ tests for demographic categorical variables. For all data analyses, the dependent variable in the present study was level of empathy as assessed by the JSPE-S that was measured twice: at the beginning (preclinical) and at the end of the fourth year. Dependent samples t-tests and ANOVA with repeated measures were used for longitudinal analyses of pre-clinical JSPE-S vs. end of the fourth-year JSPE-S scores. Tukey post-hoc comparison tests were used to examine the differences among the three groups in variables for which one-way ANOVA tests were significant. A two-sided significance level of 0.05 was established for all analyses. Data analysis was performed using Statistical Package for Social Sciences (SPSS) software, Version 26.0 for Windows.

## Results

### Demographic characteristics

The demographic characteristics of participants are presented in Table 1. There were no differences between the three cohorts in age, marital status or religiosity. Significant differences between cohorts were found in gender and ethnic origin (Table 1).

**Table 1:** Demographic characteristics of participants in the study

Cohort	PI/MH_**(lim)**_	MMI/MH_**(lim)**_	MMI/MH_(ext**)**_
Preclinical Medical Humanities program	limited MH	limited MH	extended MH
Admission system	Personal Interview	Multiple Mini Interviews	Multiple Mini Interviews
	***n*** = 91	***n*** = 86	***n*** = 85
***Gender***^**a**^			
Men	59 (64.8%)	50 (58.1%)	38 (45.2%)
Women	32 (35.2%)	36 (41.9%)	46 (54.8%)
***Age***	25.98 ± 3.52	26.10 ± 2.60	25.57 ± 3.56
***Marital Status***			
Single	67 (73.6%)	62 (75.6%)	63 (75.0%)
Married	24 (26.4%)	20 (24.4%)	21 (25.0%)
***Religiosity***			
Secular	46 (51.7%)	47 (59.5%)	43 (54.4%)
Traditional	13 (14.6%)	10 (12.7%)	12 (15.2%)
Religious	30 (33.7%)	22 (27.8%)	24 (30.4%)
***Ethnicity***^**b**^			
Jew	73 (81.1%)	78 (96.3%)	71 (87.7%)
Arab	17 (18.9%)	3 (3.7%)	10 (12.3%)

^a^The proportion of women was about one third in the PI/MH_(lim)_ cohort increasing to more than a half in the MMI/MH_(ext)_ cohort (χ^2^_(2)_ = 6.99; *p* = 0.030)

^b^A significant difference in distribution of ethnic origin between cohorts were observed (χ^2^_(2)_ = 9.40; *p* = 0.009)

### Establishing JSPE-S validity evidence

We examined convergent validity of JSPE with the Interpersonal Reactivity Index (IRI). The correlation between the JSPE-S and the IRI total score administered at the beginning of the study was *r* = 0.31; *p* < 0.001. The correlations between the JSPE-S and the IRI subscales were *r* = 0.43; *p* < 0.01 for Perspective Taking, and *r* = 0.30; *p* < 0.05 for Empathic Concern, and no correlation with the Fantasy and Personal Distress subscales, similarly to Hojat’s findings in the original JSPE English version [31].

In addition, the alpha Cronbach coefficient of internal reliability of the JSPE-S was excellent: α = 0.86 for pre-clinical JSPE-S scores, and α = 0.90 at the end of the fourth-year.

### Overall changes in JSPE-S among all subjects during the fourth-year

There were no significant differences in preclinical JSPE-S by gender, marital status, ethnicity, religiosity or residency preferences, and admission system (Table 2).

**Table 2:** Comparisons between pre-clinical and end of the fourth-year JSPE-S scores according to demographic and baseline characteristics

	Preclinical	End 4th year	***t***	***df***	***p***	***Cohen’s d***
**All students**	114.40 ± 11.32	112.75 ± 14.19	2.14	226	0.034	0.13
***Gender***						
Men	114.54 ± 11.33	112.13 ± 13.99	2.33	129	0.021	0.19
Women	114.11 ± 11.38	113.78 ± 14.42	0.29	95	0.769	0.03
***Marital status***						
Single	114.63 ± 11.08	113.26 ± 14.17	1.52	168	0.130	0.11
Married	114.02 ± 12.23	112.43 ± 13.97	1.09	53	0.282	0.12
***Ethnicity***						
Jew	114.39 ± 11.53	112.94 ± 13.97	1.76	193	0.079	0.11
Arab	114.65 ± 10.31	113.29 ± 14.91	0.58	24	0.565	0.10
***Religiosity***						
Secular	113.55 ± 12.14	112.44 ± 13.53	1.08	118	0.282	0.09
Traditional	115.50 ± 10.13	112.52 ± 17.36	1.26	30	0.218	0.19
Religious	115.23 ± 10.95	115.00 ± 11.93	0.21	64	0.837	0.02
***Admission system***						
Personal Interview	112.91 ± 12.35	110.98 ± 14.54	1.59	81	0.115	0.18
Multiple mini Interviews	115.24 ± 10.64	113.75 ± 13.94	1.49	144	0.137	0.12
***Residency preferences***						
Surgical residency	113.87 ± 11.41	108.96 ± 17.04	2.02	38	0.050	0.33
Non- surgical residencies	114.50 ± 11.26	113.77 ± 13.39	0.92	178	0.359	0.06

Among all subjects, there was a small but significant decrease in JSPE-S during the fourth-year of studies (114.40 ± 11.32 vs. 112.75 ± 14.19, *t*_(226)_ = 2.14, *p* = 0.034, Table 2). Among men from the three cohorts, but not among women, JSPE-S scores declined significantly during the fourth-year (In men: 114.54 ± 11.33 vs 112.13 ± 13.99, *t*_(129)_ = 2.33, *p* = 0.021, in women: 114.11 ± 11.38 vs 113.78 ± 14.42, *t*_(95)_ = 0.77, *p* = 0.769,Table 2). Since the decline in JSPE-S was observed in men but not in women, data analysis is presented according to gender (see below).

### The effect of MH program on JSPE-S change

In order to evaluate the effect of MH program (limited vs extended) and time (beginning and end of year), and their interaction on JSPE-S, repeated measures ANOVA was performed. The analysis included JSPE-S scores as dependent variable, and MH program and time as independent measures. In order to control for admission system, it was added to the initial analysis as a covariate. Since it did not have a significant contribution, we did not include admission system in the final analysis.

**Fig. 2:**
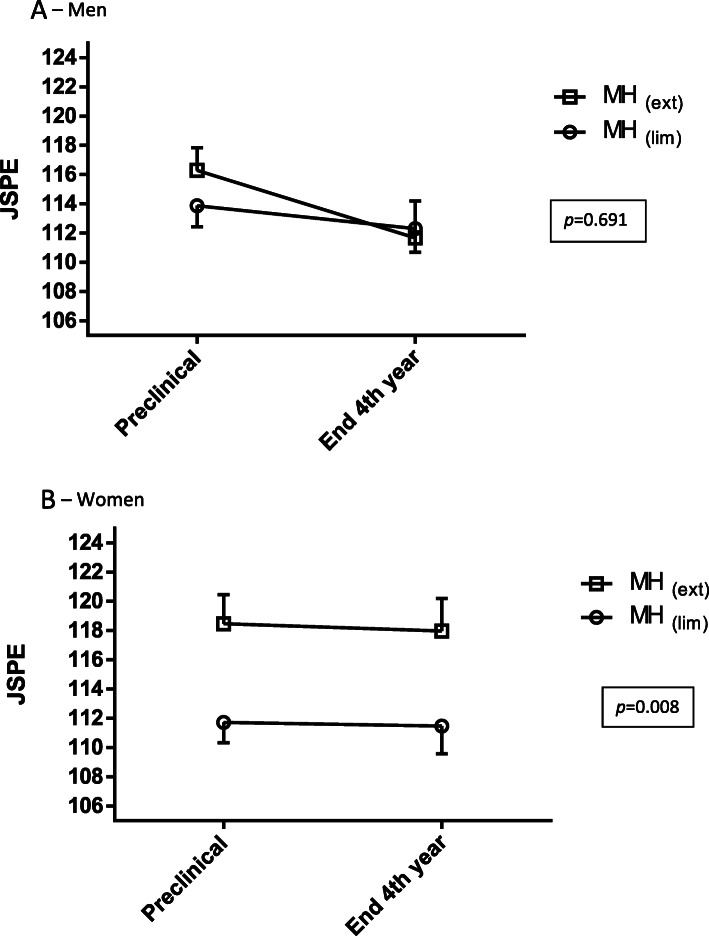
The effect of medical humanities curriculum on JSPE-S during the fourth-year (Mean ± SEM) among men (Panel 1A) and women (Panel 1B), [*p* values are for the main effect of medical humanities curriculum on JSPE-S scores, two-way ANOVA of JSPE-S by time (pre-clinical – end of the 4th year) and by humanities program, performed separately in men and women]. Panel 2A – Men. *MH*_*(lim*)_: limited Medical Humanities program, *MH*_*(ext)*_: extended three-year Medical Humanities studies. Panel 2B – Women. *MH*_*(lim)*_: limited Medical Humanities program, *MH*_*(ext)*_: extended three-year Medical Humanities studies

Among men there was no effect of MH on JSPE-S scores (*F*_(1,128)_ = 0.16, *p* = 0.691), there was a significant effect of time on JSPE-S (*F*_(1,128)_ = 7.26, *p* = 0.008), and there was no interaction between humanities program and time (*F*_(1,128)_ = 1.78, *p* = 0.185; Table 3, Fig. 2 – Panel 2A). However, among men students of the MH_(ext)_ cohort we found a significant decline in JSPE-S during the fourth-year of studies (*t*_(35)_ = 2.38, *p* = 0.023; Table 3), while no significant decline was observed in the MH (_lim_) cohort (*t*_(93)_ = 1.29, *p* = 0.200; Table 3).

**Table 3:** Comparisons between preclinical and end of the fourth-year JSPE-S scores by Medical Humanities program and gender

	Preclinical	End 4th year	***t***	***df***	***p***	***Cohen’s d***
***Men***						
MH_(lim)_	113.87 ± 11.99	112.31 ± 13.59	1.29	93	0.200	0.13
MH_(ext)_	116.29 ± 9.30	111.67 ± 15.19	2.38	35	0.023	0.34
***Women***						
MH_(lim)_	111.72 ± 10.69	111.48 ± 14.81	0.16	61	.871	0.02
MH_(ext)_	118.47 ± 11.43 **	117.97 ± 12.86*	0.29	33	0.775	0.04

*Humanities program – MH*_*(lim)*_ Limited Medical Humanities program*, MH*_*(ext)*_ Extended three-year Medical Humanities studies

*p < 0.05 for difference between *MH*_*(lim)*_ and MH_(ext)_ in preclinical JSPE-S in women

**p < 0.01 for difference between *MH*_*(lim)*_ and MH_(ext)_ in end of 4th-year JSPE-S in women

Among women, MH was significantly associated with JSPE-S scores (*F*_(1,94)_ = 7.46, *p* = 0.008). However, there was no effect for time (*F*_(1,94)_ = 0.10, *p* = 0.756) and no interaction between MH program and time (*F*_(1,64)_ = 0.012, *p* = 0.914; Table 3, Fig. 2 – Panel 2B). These findings indicate that women who participated in MH_(ext)_ as compared to MH_(lim)_ showed higher JSPE-S scores, and that women who participated in either MH_(ext)_ or MH_(lim)_ did not show a decline in JSPE-S (Table 3).

In order to evaluate if the impact of MH_(ext)_ on JSPE-S was not only statistically significant but also substantial, we calculated Cohen’s *d* effect sizes: At baseline, differences in empathy scores between women who participated in MH_(ext)_ and those who did not were significant (*t*_(94)_ = 2.89, *p* = .005), yielding a large effect size: Cohen’s *d* = .61. At the end of the fourth-year, differences between the two groups of women were still significant (*t*_(94)_ = 2.15, *p* = .034), yielding a medium effect size: Cohen’s *d* = .47.

## Discussion

The main findings of this study are that empathy declined among men medical students’ during their first extensive clinical experience, and that the decline was associated with pre-clinical humanities program. Among women students, there was no decline in empathy during the fourth-year of studies, regardless of type of humanities program.

In addition, women who participated in the extended humanities program had higher JSPE-S scores during the fourth-year as compared to women who participated the limited program.

In contrast to the finding in women, among men we found a significant decline in empathy during the fourth-year of studies in those who participated in the extended humanities program, but not in those who participated in the limited program.

Our study suggests, according to the large to medium effect sizes observed, that the differences in empathy scores in women who participated in the extended humanities program as compared to the limited program are not only statistically significant but are also likely to be substantial. Further study is required to determine the practical and educational implications of these findings. Such implications may include increased awareness of curriculum planners to potential sources for gender differences in educational interventions aimed to enhance empathy among medical students.

Previous studies [1, 10–12] have suggested that an overall decline in JSPE-S scores during the third-year of medical studies exists. Our study suggests that an extensive program that included exposure to ethical issues, communication skills, and humanities studies, was associated with a decline in empathy among men students, while among female students a decline was not observed, regardless of medical humanities program.

Previous studies on gender effect on medical students’ empathy yielded inconsistent findings [1, 11, 15–17]. Our findings suggest that gender differences in empathy may exist, and go along with gender differences previously reported in clinical practice [32, 33]. It is possible that gender-specific impact of educational programs that we and others [22, 23] have observed were underestimated in previous studies because of small samples which did not allow to assess such effects. It is also possible that measuring empathy at a single time point would be less sensitive to detect gender differences in empathy as compared to longitudinal studies.

A secondary aim of the study was to contribute to the validity evidence of the Hebrew version of the JSPE-S by examining its relationship with an established measure of empathy. We observed a significant correlation between the JSPE-S and the IRI total score administered at the beginning of the study. The correlations between the JSPE-S and the IRI subscales were significant for Perspective Taking and for Empathic Concern subscales, while no correlation was observed with the Fantasy and Personal Distress subscales, similarly to the findings of Hojat in the original JSPE English version [31], providing validity evidence of the Hebrew version of the JSPE-S.

Because our study was not randomized it is possible that the observed differences in empathy change between cohorts are the result of baseline difference between cohorts. Although there was no difference between cohorts in gender, average age, marital status, it is possible that other variables that were not captured by sociodemographic measures that were collected contributed to the observed differences.

The decline in empathy during the first clinical year, while students are introduced to the clinical work in the wards, can have several potential explanations. These include de-idealization of students’ perception of medicine [34], lack of proper role models [35], and students’ perception that, as compared to the power of technology and the intense clinical experience, empathy may not be a significant tool in the profession of medicine as students had believed it to be before they entered clinical life [36]. Students can easily put aside the importance of interpersonal engagement in patient care when the majority of their studies are based on quantitative scientific outcomes. At the same time, the decline in empathy among medical students may reflect a protective mechanism that can help students to deal with emotionally difficult situations [10].

Counterintuitively, the larger decline in empathy was observed in male students from the cohort who participated in the extended medical humanities program, as compared to male students who participated in the limited program.

This finding may be explained by the lack of continuous medical humanities program during the fourth year of studies. This could have potentially resulted in the greater decline in empathy in students who were used to participate regularly in a medical humanities program during their first 3 years of studies, and had a greater level of empathy at the beginning of the fourth year, as compared to students who received only a limited program during their first year of studies (even though the difference at the beginning of the fourth year was not statistically significant). At the end of the fourth year, empathy in both cohorts was similar, but the decline was significantly greater in the extended medical humanities cohort, who had higher levels of empathy at the beginning of the fourth year of studies.

These findings do not support the possibility that extensive pre—clinical medical humanities programs have an “immunizing” effect on medical students’ decline in empathy during the clinical studies, and may support the need for continuous medical humanities program through-out medical studies.

Additional research is required to investigate this possibility, and to determine why the decline was observed in men but not in women.

Our study has several limitations. The study included a single medical school, which may limit the generalization of the findings. Cultural differences and differences in the average age in which students start medical school, may affect students’ previous life experiences and empathy levels. For example, the average starting age for medical school in Israel is higher than in USA [37] or Ethiopia [38]. Such differences might have an impact on our results regarding students’ empathy. In addition, our study was based on a self-reported empathy measurement, the JSPE-S, and not on observed behaviors, that may only partially correlate [39, 40].

We followed students during the 4th year of studies, and not during all 3 clinical years. This has likely limited our conclusions regarding changes in empathy during medical studies. In addition, the aim of this study was to explore empathy changes that have been previously suggested to occur following the first students’ clinical experiences during clerkships. Thus, we evaluated empathy at the beginning and following the fourth year of medical studies in three consecutive classes of medical school. However, since JSPE-S scores at entry to medical school are not available, we cannot exclude the possibility that differences in empathy between cohorts, prior to entrance to medical school, could have contributed to our findings. However, we examined the effect of medical humanities curriculum on empathy in two cohorts of students that were admitted to medical school using the same admission system (MMI), and this could contribute to reduce differences between cohorts in baseline empathy.

Due to the observational design of our study, students’ randomization to the medical humanities programs was not possible, and comparisons were made between cohorts. Although randomization is considered a gold standard in clinical studies, it has been recognized that it is difficult to blind learners to their assigned group in educational studies [41]. A clinical research model that has been suggested to be more applicable for educators is the “pragmatic trail” in which interventions are compared in real-world practice [41]. Thus, we measured empathy in a prospective controlled study. We used a longitudinal design with repeated measurements to compare changes in empathy over time in the cohorts studied. In addition, a single humanities program was offered in each year (limited/extended), and students were obligated to participate in it, therefore students’ preferences could not affect their participation in the limited/extended programs.

## Conclusions

In women, empathy did not decline during the fourth year of medical school, while in men the decline in empathy during the fourth year was associated with the extensive medical humanities program. These findings suggest that extensive pre—clinical medical humanities program did not have an “immunizing” effect on a decline in empathy in men students’ during the clinical studies.

Our findings regarding gender-specific effects of medical humanities program require further validation. Such research may help to design continuous educational interventions to address the decline in empathy in men and women students during the course of medical studies.

## Data Availability

The datasets used and/or analyzed during the current study are available from the corresponding author on reasonable request.

## Abbreviations

PI: Personal interview
MMI: Multiple mini interviews
MH_(lim)_: Limited Medical Humanities program
MH_(ext)_: Extended Medical Humanities program

## References

[1] Hojat M, Vergare MJ, Maxwell K, Brainard G, Herrine SK, Isenberg GA, Veloski J, Gonella JS (2009). The devil is in the third year: a longitudinal study of erosion of empathy in medical school. Acad Med.

[2] Brownell AK, Côté L (2001). Senior residents’ views on the meaning of professionalism and how they learn about it. Acad Med.

[3] Hojat M, Gonnella JS, Nasca TJ, Mangione S, Vergare M, Magee M (2002). Physician empathy: definition, components, measurement, and relationship to gender and specialty. Am J Psychiatry.

[4] Davis MH (1983). Measuring individual differences in empathy: evidence for a multidimensional approach. J Pers Soc Psychol.

[5] Hojat M, Mangione S, Nasca TJ, Cohen MJM, Gonnella JS, Erdmann JB, Veloski J, Magee M (2001). The Jefferson scale of physician empathy: development and preliminary psychometric data. Educ Psychol Meas.

[6] Mercer SW, Reynolds WJ (2002). Empathy and quality of care. Br J Gen Pract.

[7] Kim SS, Kaplowitz S, Johnston MV (2004). The effects of physician empathy on patient satisfaction and compliance. Eval Health Prof.

[8] Vermeire E, Hearnshaw H, Van Royen P, Denekens J (2001). Patient adherence to treatment: three decades of research. A comprehensive review. J Clin Pharm Ther.

[9] Levinson W, Roter DL, Mullooly JP, Dull VT, Frankel RM (1997). Physician-patient communication: the relationship with malpractice claims among primary care physicians and surgeons. JAMA..

[10] Chen D, Lew R, Hershman W, Orlander J (2007). A cross-sectional measurement of medical student empathy. J Gen Intern Med.

[11] Newton BW, Barber L, Clardy J, Cleveland E, O'Sullivan P (2008). Is there hardening of the heart during medical school?. Acad Med.

[12] Hojat M, Mangione S, Nasca TJ, Rattner S, Erdmann JB, Gonnella JS, Magee M (2004). An empirical study of decline in empathy in medical school. Med Educ.

[13] Neumann M, Edelhäuser F, Tauschel D, Fischer MR, Wirtz M, Woopen C, Haramati A, Scheffer C (2011). Empathy decline and its reasons: a systematic review of studies with medical students and residents. Acad Med.

[14] Colliver J, Conlee M, Verhulst S, Dorsey L (2010). Reports of the decline of empathy during medical education are greatly exaggerated: a reexamination of the research. Acad Med.

[15] Chen D, Kirshenbaum D, Yan J, Kirshenbaum E, Aseltine R (2012). Characterizing changes in student empathy throughout medical school. Med Teach.

[16] Newton BW, Savidge MA, Barber L, Cleveland E, Clardy J, Beeman G, Hart T (2000). Differences in medical students’ empathy. Acad Med.

[17] Hojat M, Gonnella JS, Mangione S, Nasca TJ, Veloski JJ, Erdmann JB, Callahan CA, Magee M (2002). Empathy in medical students as related to academic performance, clinical competence and gender. Med Educ.

[18] Hojat M, Axelrod D, Spandorfer J, Mangione S (2013). Enhancing and sustaining empathy in medical students. Med. Teach..

[19] Rosenthal S, Howard B, Schlussel YR, Herrigel D, Smolarz BG, Gable B, Vasquez J, Grigo H, Kaufman M (2011). Humanism at heart: preserving empathy in third-year medical students. Acad Med.

[20] Muszkat M, Ben-Yehuda A, Moses S, Naparstek Y (2010). Teaching empathy through poetry: a clinically based model. Med Educ.

[21] Muszkat M, Barak O, Lalazar G, Mazal B, Schneider R, Mor-Yosef Levi I, Cohen MJ, Canetti L, Ben Yehuda A, Naparstek Y (2014). The effect of medical students’ gender, ethnicity and attitude towards poetry-reading on the evaluation of a required, clinically-integrated poetry-based educational intervention. BMC Med Educ.

[22] Shapiro J, Morrison E, Boker J (2004). Teaching empathy to first year medical students: evaluation of an elective literature and medicine course. Educ Health.

[23] Kommalage M (2011). Using videos to introduce clinical material: effects on empathy. Med Educ.

[24] Eva KW, Rosenfeld J, Reiter HI, Norman GR (2004). An admissions OSCE: the multiple mini-interview. Med Educ.

[25] Shaham D, Kandel L, Gural A (2011). Establishing a medical humanities program in Israel: challenges and solutions. Eur Leg.

[26] Karniol R, Gabay R, Ochion Y, Harari Y (1998). Is gender or gender-role orientation a better predictor of empathy in adolescence. Sex Roles.

[27] Gabay Y, Shamay-Tsoory SG, Goldfarb L (2016). Cognitive and emotional empathy in typical and impaired readers and its relationship to reading competence. J Clin Exp Neuropsychol.

[28] Uzefovsky F, Shalev I, Israel S, Edelman S, Raz Y, Mankuta D, Knafo-Noam A, Ebstein RP (2015). Oxytocin receptor and vasopressin receptor 1 a genes are respectively associated with emotional and cognitive empathy. Horm Behav.

[29] Di Lillo M, Cicchetti A, Scalzo AL, Taroni F, Hojat M (2009). The Jefferson scale of physician empathy: preliminary psychometrics and group comparisons in Italian physicians. Acad Med.

[30] Kliszcz J, Nowicka-Sauer K, Trzeciak B, Nowak P, Sadowska A (2006). Empathy in health care providers-validation study of the polish version of the Jefferson scale of empathy. Adv Med Sci.

[31] Hojat M, Mangione S, Kane GC, Gonnella JS (2005). Relationships between scores of the Jefferson scale of physician empathy (JSPE) and the interpersonal reactivity index (IRI). Med. Teach..

[32] Bensing JM, van den Brink-Muinen A, de Bakker DH (1993). Gender differences in practice style: a Dutch study of general practitioners. Med Care.

[33] Bertakis KD, Helms LJ, Callahan EJ, Azari R, Robbins JA (1995). The influence of gender on physician practice style. Med Care.

[34] Kay J (1990). Traumatic deidealization and the future of medicine. JAMA..

[35] Kenny NP, Mann KV, MacLeod H (2003). Role modeling in physicians’ professional formation: reconsidering an essential but untapped educational strategy. Acad Med.

[36] Griffith CH, Wilson JF (2001). The loss of student idealism in the 3rd-year clinical clerkships. Eval. Health Prof..

[37] Austin EJ, Evans P, Goldwater R, Potter V (2005). A preliminary study of emotional intelligence, empathy and exam performance in first year medical students. Pers Individ Dif.

[38] Dehning S, Gasperi S, Tesfaye M, Girma E, Meyer S, Krahl W, Riedel M, Möller H-J, Müller N, Siebeck M (2013). Empathy without borders? Cross-cultural heart and mind-reading in first-year medical students. Ethiop J Health Sci.

[39] Hojat M, Mangione S, Nasca TJ, Gonnella JS, Magee M (2005). Empathy scores in medical school and ratings of empathetic behavior in residency training 3 years later. J Soc Psychol.

[40] Colliver JA, Willis M, Robbs RS, Cohen DS, Swartz MH (1998). Assessment of empathy in a standardized-patient examination. Teach Learn Med.

[41] Sullivan GM (2011). Getting off the “gold standard”: randomized controlled trials and education research. J Grad Med Educ.

